# Supplementary material for: A hierarchy of cell death pathways confers layered resistance to shigellosis in mice
Source: eLife. 2023 Jan 16;12:e83639. doi: 10.7554/eLife.83639 (PMC9876568; doi:10.7554/eLife.83639)

LI-COR®

**Image ID: 0000809_01**
**Acquire Time: Nov 17, 2020 2:03:48 PM**



Acquisition Information

| # | Image ID | Acquire Time | Channels | Resolution | Intensities | Quality | Analysis | Image Name | Comment |
|---|---|---|---|---|---|---|---|---|---|
| 1 | 0000809_01 | Nov 17, 2020 2:03:48 PM | 700 | 169um | Auto | lowest | Manual | 0000809_01 | |

Image Display Values

| Channel | Color | Minimum | Maximum | K |
|---|---|---|---|---|
| 700 | Gray Scale (Black on White) | 1.18 | 6.82 | 0 |

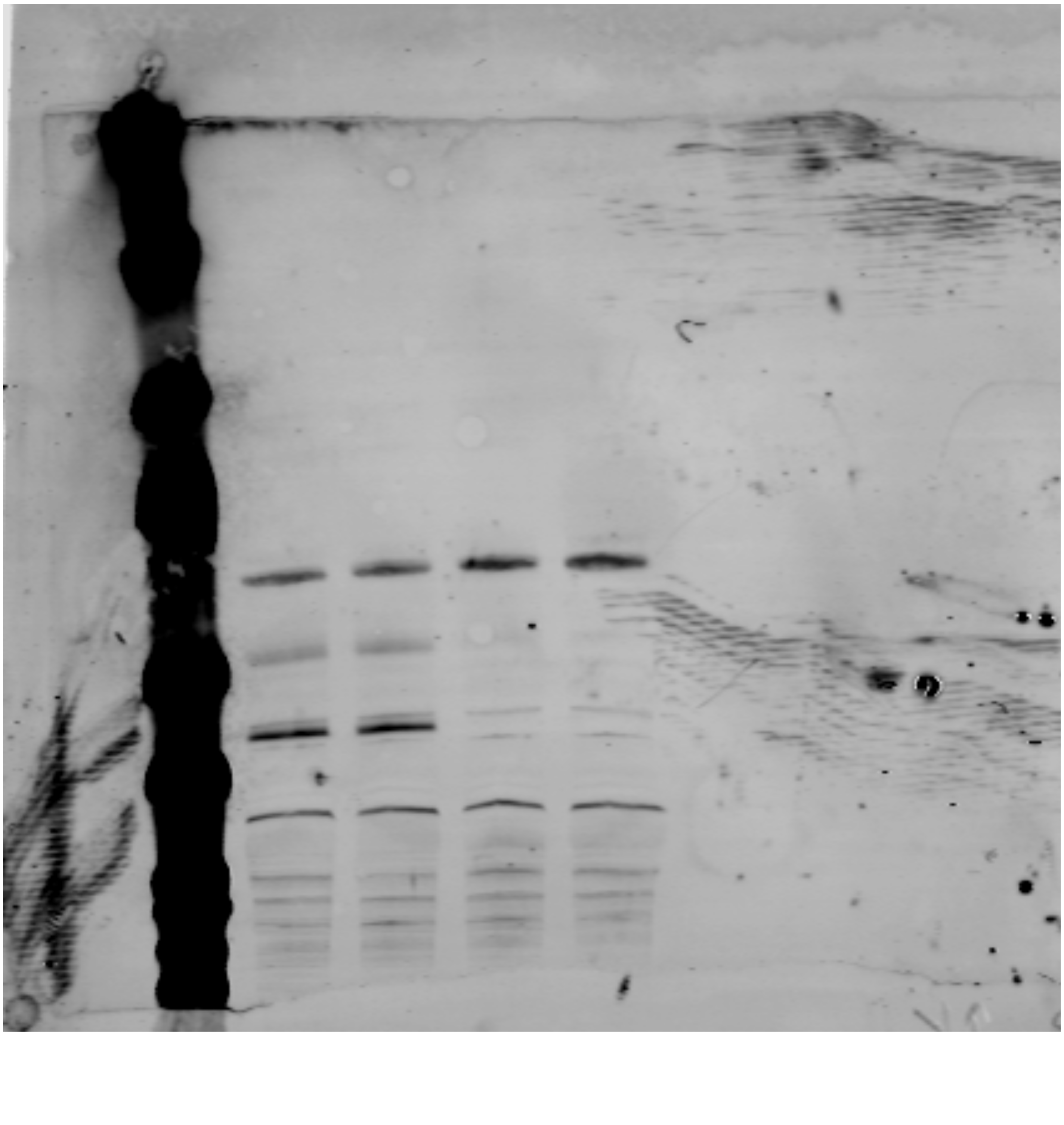

Supplement: Figure 2—figure supplement 1—source data 1. — Raw images were cropped and inverted to show lanes in a more readable orientation. [file elife-83639-fig2-figsupp1-data1.zip › Figure 2 - figure supplement 1 - source data 1/Casp11KO_rawblot.pdf]
