# Supplementary figures and images for: A hierarchy of cell death pathways confers layered resistance to shigellosis in mice
Source: eLife. 2023 Jan 16;12:e83639. doi: 10.7554/eLife.83639 (PMC9876568; doi:10.7554/eLife.83639)

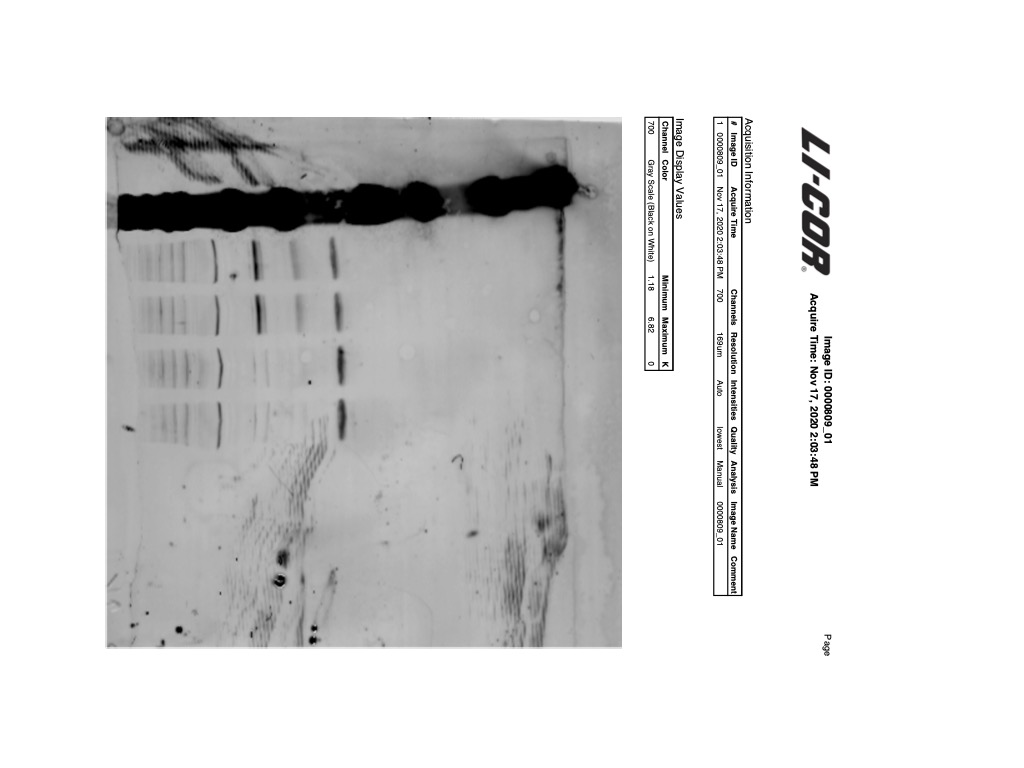

Supplement: Figure 2—figure supplement 1—source data 1. — Raw images were cropped and inverted to show lanes in a more readable orientation. [file elife-83639-fig2-figsupp1-data1.zip › Figure 2 - figure supplement 1 - source data 1/Casp11KO_rawblot.jpg]

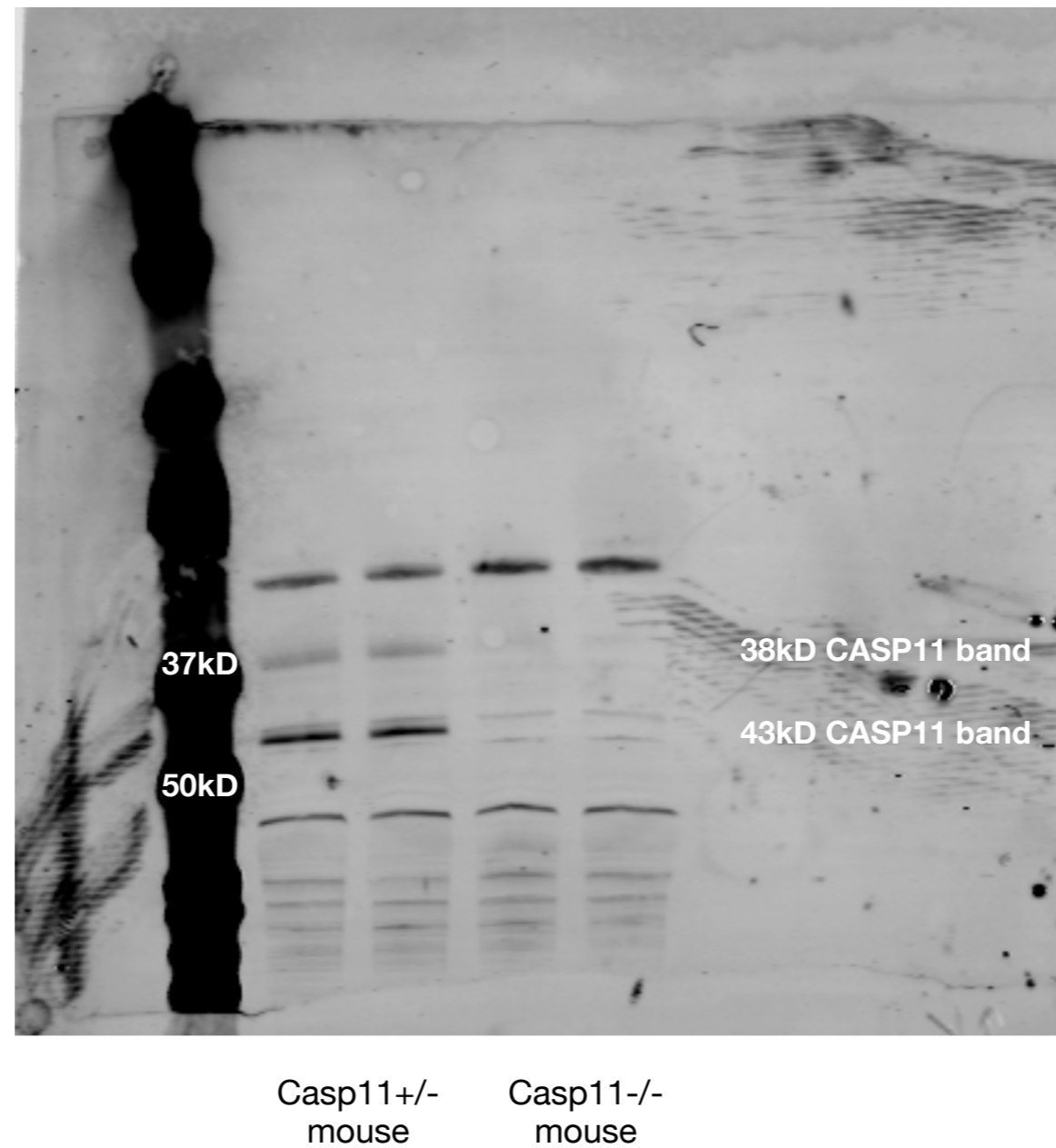

(Gel is inverted vertically in figure 2 – figure supplement 1 for more readable orientation)

Supplement: Figure 2—figure supplement 1—source data 1. — Raw images were cropped and inverted to show lanes in a more readable orientation. [file elife-83639-fig2-figsupp1-data1.zip › Figure 2 - figure supplement 1 - source data 1/Casp11KO_labeleduncroppedblot.pdf]
